# Increasing participation in research with therapy dogs: A qualitative study at a large urban mental health and addiction hospital

**DOI:** 10.1371/journal.pone.0238096

**Published:** 2020-08-27

**Authors:** Laura Sikstrom, Tamar Meyer, Eva Katz, Man-Man Choi, Margaret Darragh, Amanda Cutler-Palma, Theresa Conforti, Csilla Kalocsai, Sophie Soklaridis

**Affiliations:** 1 Office of Education, Centre for Addiction and Mental Health, Toronto, Ontario, Canada; 2 Geriatric Mental Health Services, Centre for Addiction and Mental Health, Toronto, Ontario, Canada; 3 Complex Care & Recovery Program, Centre for Addiction and Mental Health, Toronto, Ontario, Canada; 4 Volunteer Services, Centre for Addiction and Mental Health, Toronto, Ontario, Canada; 5 Departments of Psychiatry and Family and Community Medicine, University of Toronto, Toronto, Ontario, Canada; 6 Wilson Centre, University Health Network and Faculty of Medicine, University of Toronto, Toronto, Ontario, Canada; University of Auckland, NEW ZEALAND

## Abstract

The benefits of involving patients as partners in research across diverse medical and psychiatric settings are well established in the literature. However, researchers continue to struggle to access, engage and retain participants from hard-to-reach populations. The main objective of this study was to co-create pet therapy activities with patients admitted for serious and complex mental illness to a large urban mental health and addiction hospital. Informed by the principles of participatory action research methodology, we conducted focus group discussions with 38 inpatients in seven different clinical units. An experienced volunteer handler and a certified therapy dog helped facilitate our discussions. Participating researchers, recreational therapists, volunteer handlers and our participants all reported that the presence of a certified therapy dog at each of our discussions was integral to their success. Certified therapy dogs increased the *motivation* to participate in our study, helped to build *rapport* with participants and created *connections* in our discussions that enriched our data. To our knowledge our study is the first to demonstrate the value of using a therapy dog as a participatory research tool in a healthcare setting. The authors believe that therapy dogs are a low-tech intervention that could be used effectively to engage hard-to-reach populations in research about their treatment and care in a diverse range of medical settings. These findings support the creation of a pilot study to test the value of including therapy dogs in patient-centered research with vulnerable and hard-to-reach populations.

## Introduction

The benefits of involving patients as partners in research across diverse medical and psychiatric settings are well established in the literature. [1] However, the failure to recruit, engage and retain hard-to-reach populations limits the scope of these efforts. [2,3,4] Significantly, unequal representation in research undermines patient outcomes for vulnerable and disadvantaged groups. [5] Moreover many qualitative research techniques rely on the desire and ability of the patient to effectively communicate their perspectives to researchers, which can be difficult in the midst of a mental health crisis. [6,7] While a range of techniques have been developed to recruit and engage patients in health research, to our knowledge our study is the first to demonstrate the value of using a certified therapy dog as a participatory research tool in a healthcare setting. [8] Somewhat unexpectedly we found that therapy dogs buffer the power and communication gaps between researchers and patients hospitalized with complex and serious mental illness. Therapy dogs increased the *motivation* to participate in our study, helped to build *rapport* with participants and created *connections* that enriched our data. Although high-tech innovations are revolutionizing healthcare and patient outcomes, our study provides evidence that therapy dogs are a “no-tech” and low cost intervention that both humanizes the hospital experience and significantly improve efforts to engage patients in research.

In recent years, the benefits of animal-human interactions, particularly with dogs, across diverse medical and psychiatric settings has been well established in the literature. Canine Assisted Interventions (CAI) bring together credentialed canine-human teams to enhance human well-being. [9] There is growing evidence on the positive impact of animal-human interactions. For example, it has been documented to reduce cardiovascular stress, [10] enhance immune factors, [11] decrease pain, [12] improve mood, [13] reduce cortisol levels, [14] decrease fear and anxiety, [15] and create an overall humanizing atmosphere within a hospital setting. [16] Specific to mental health, a meta-analysis of the effects of CAI on depression identified effects of medium magnitude. [17] A literature review concludes that CAI may also ameliorate the behavioral and psychological symptoms of dementia. [18] However, there is no available literature on CAI initiatives that have been designed *with* input from patients. Recently, a move towards building opportunities for greater collaboration with patients within the mental health and addictions system has occurred. Thus, our exploratory qualitative study was designed to emphasize the value of the patient voice within clinical settings. [19] Our aim was to co-develop our CAI activities (herein referred to colloquially as “Pet Therapy”) with patients at the Centre for Addiction and Mental Health (CAMH), which is North America’s largest mental health and addiction hospital. Although we were successful at co-creating meaningful activities with patients, this is not the focus of this article. Rather our aim is to illustrate the value of including a therapy dog in qualitative research activities with hard-to-reach populations.

### Approach

#### Setting

CAMH has more than 34,000 unique patients per year. CAMH’s “Pet Therapy” consists of 40 dogs and their volunteer handlers. Dogs are screened, tested and evaluated before they can be part of CAMH’s Pet Therapy program. On average 70% of the dogs pass the assessment, which focuses on ascertaining a dog’s response to unpredictable situations, commands, loud noises and strangers. Once the dogs are evaluated and pass, the volunteer handlers are interviewed, trained and placed in a specific clinical unit. Volunteers visit one to two units per week for 30 minutes to one hour at a time. Pet Therapy activities are highly variable across the hospital but typically involve an informal gathering of interested patients who pet, play and chat with the visiting dog and handler. There are also one-on-one visits and more informal gatherings at the library. Pet Therapy is a very popular program with patients and staff. All of the dogs used in this study have been tested and certified to work in clinical settings and were accompanied by their handler the entire time.

#### Methods

The findings presented here are part of an exploratory patient engagement project at CAMH on Canine Assisted Therapy activities. Our research question was: How can patients partner with the volunteer handlers and dogs at CAMH to co-create activities that are meaningful to them, improve their recovery process and humanize the psychiatric hospital experience?

To engage patients at CAMH as research partners our methodology was informed by the principles of Participatory Action Research (PAR). PAR differs from other methods of mental health research due to its focus on reflection, data collection, and action that works to improve health and reduce health inequities by involving the individuals affected. [20] One benefit of PAR is that researchers often design the research questions and methodology with potential participants to ensure that the questions are considered both relevant and beneficial to the population being studied. [21] In our study, one strategy we used was to work with volunteer handlers to refine our discussion guide. A second (unintentional) strategy was to bring pet therapy dogs to all our research activities. The impact of this decision on our research is the focus of this article. Ethics approval for this study was granted by the Research Ethics Board at CAMH.

Our recruitment strategy involved a five step process between September and December 2018. First, we sent a recruitment email to all of the pet therapy volunteer handlers at CAMH asking if they would like to co-facilitate a focus group discussion (FGD) on pet therapy with patients. Seven handlers expressed immediate interest. These handlers had a combined total of 20 years of experience conducting pet therapy visits on clinical units at CAMH. This meant that in some cases they had established relationships with some of the participants in our study. Second, we reached out to the recreational therapist (RT) on the clinical units where these handlers volunteered to describe the aims of the study and obtained their permission to hold a FGD on their unit. All of the units we approached agreed to participate in our study. Third, all of the volunteer handlers participated in a webinar on PAR methodology and facilitation skills. At this stage we consulted with the handlers to determine whether they felt able to co-facilitate a FGD while also monitoring their dog’s interactions with patients. Handlers decided unanimously that they would like to bring their dogs to each FGD.

Fourth, handlers, RTs and researchers informed inpatients of our study face-to-face during a community meeting held on each clinical unit. A researcher [LS] and volunteer handlers also informed patients of the study in person during regularly scheduled pet therapy visits held on these clinical units. In addition to communicating the aims of our study in person we also distributed a letter of information for those interested in learning more about our study. Finally, patients expressed their interest in participating to the RT, who then coordinated the best time and place to hold our discussion. All potential participants were informed of the aims of the study and that their participation was voluntary. Written informed consent was obtained from all participants on the day of the scheduled FGD. All of our participants were also informed that their participation (or lack thereof) would have no effect on their care. One patient declined to participate after expressing initial interest but did not provide a reason for his refusal.

We held a total of seven FGD with 38 patients (18 men, 20 women). We conducted our FGD on seven of the 23 clinical units at CAMH that offer pet therapy to patients (see Table 1). All of our participants had been admitted to a clinical unit at CAMH for a minimum of 72 hours and up to 4 years. Two of our FGD took place on forensics unit, thus our participants may disproportionately represent those with mental illness that have had encounters with the law. Our participants ranged in age from 18 to 88years. We did not ask any questions about their personal medical histories. We did not witness any signs of distress in the dogs during our FGD. The dogs appeared happy and relaxed and moved from patient to patient seeking pets and dog treats. However, handlers did report that their dogs occasionally demonstrated signs of distress on units during official pet therapy visits. For example, on one unit an acute patient became very loud and aggressive and the dog indicated that they wanted to leave by heading towards the exit.

**Table 1:** Clinical unit descriptions and participant Information by Gender.

*Clinical Unit*	*Description*	*Men*	*Women*	*Total*
Women’s Inpatient Unit [WIU]	A women’s only inpatient unit. Patients are admitted for a range of conditions from mood disorders to self-harm and addiction. Most patients have unaccompanied passes off the unit. Length of stay is approximately 6 weeks.		7	7
Geriatric Admission Unit—A [GAU-A]	An inpatient unit with an emphasis on concurrent disorders in elder care (e.g. dementia and schizophrenia or dementia and addiction). There are two outdoor spaces attached to the unit. A number of patients have unaccompanied passes off the unit if they show they can keep time, can find their way back independently, are not in distress and are cooperative with treatment. Length of stay varies from 2 weeks-4 years due to a backlog for supportive housing and long term care.	3	1	4
Geriatric Admission Unit B—[GAU-B]	An inpatient unit with an emphasis on dementia care, schizophrenia, mood disorders and addiction. There are two outdoor spaces attached to the unit. A number of patients have unaccompanied passes off the unit if they show they can keep time, can find their way back independently, are not in distress and are cooperative with treatment. Length of stay varies from 2 weeks-4 years due to a backlog for supportive housing and long term care.	2	2	4
General Psychiatry [GPU]	General Psychiatry is the primary entry point into care from CAMH’s emergency department and is characterized by high rates of acuity. Most patients have undiagnosed conditions. Patients are referred to other units after a preliminary evaluation. Average stay is ideally only seven days but is often longer. Very few patients have unaccompanied passes.	4	5	9
Acute Care -Schizophrenia	A schizophrenia unit with high rates of acuity. Very few patients have unaccompanied passes off of the unit. The average length of stay is 3–14 days. Readmissions are common.	4		4
Women’s Secure Forensics Unit [WSFU]	One of two secure female-only forensics units in Ontario, Canada. A forensics unit is for individuals that have a mental illness and have come into contact with the law. There is a small fenced outdoor space attached to the unit. The length of stay varies from months to years.	2	2	4
Forensics	A low-medium security forensics unit for both men and women. A forensics unit is for individuals with a mental illness that have come into contact with the law. The length of stay varies from months to years.	3	3	6
**Total**		**18**	**20**	**38**

Each FGD was held in a private activity room on each clinical unit and co-facilitated by a medical anthropologist [LS] and a volunteer handler. RTs participated in three of the seven FGD due to hospital regulations about the staff supervision of patients with a recent history of violent outbursts. Any inpatient with an interest in pet therapy was eligible to participate in this study. However, in a few instances the RT identified patients that were having difficulties related to their illness (e.g. hallucinations, erratic behavior, or violent outbursts) and were excluded from participating on the day of our FGD. In addition, patients who required a substitute decision-maker to consent were excluded from this study (e.g. patients with advanced dementia). Outpatients/ambulatory patients were also excluded from this study since our study was concerned with the experience of hospitalization from the patient’s perspective. Most participants had attended pet therapy activities at least once prior to our FGD. Two of our participants had a very limited capacity to communicate verbally (e.g. one participant experiences selective mutism), however the presence of the dog helped *buffer* these communication challenges and enabled us to successfully engage the patient’s perspective of a hospital-based pet therapy program.

Each FGD lasted between 50–90 minutes. We asked participants about their previous experiences with pet therapy, what they liked best about pet therapy and if they had any negative experiences with pet therapy (see S1 Appendix A). FGDs are a useful methodological technique for many reasons but for this article we illustrate their ability to capture communication *between* research participants to generate data. [22] In particular, a medical anthropologist with ethnographic expertise [LS] took field notes during each FGD on the kinds of social interactions generated by the dog’s presence. In addition, researchers documented the main discussion points on a flip chart. In addition, at the end of each FGD we summarized the main points with our participants to check for accuracy. We did not check for accuracy after the analysis was complete (member checking) due to the transience and memory loss of many of our research participants. Using an iterative research process we reflected on these observations after each FGD to refine our discussion guide and make further observations. Thus, our findings reflect not only what was said explicitly during each FGD but also draw upon observations about how the therapy dog influenced the social interactions between patients and researchers.

Each FGD was also audio recorded and transcribed. A qualitative computer software package, Dedoose, was used to analyze and code the data. First, LS coded for general descriptive codes (e.g. meaning of pet therapy). Next, these primary descriptive codes were synthesized into axial codes. Axial coding in grounded theory is the process of relating categories and concepts to each other via inductive and deductive thinking. [23] Our axial codes reflected three major themes that emerged from both our observations and our participants’ verbal responses during the FGD: motivation, rapport and connection. Given our methodological approach, the positionality of our research team is relevant. Within our authorship team we have a mix of social scientists, recreational therapists and volunteer handlers. Our lead author (LS) for example, was a skeptic. She had no previous experience with pet therapy, or dogs, and her surprise at the ability of certified therapy dogs to enrich our data, was the impetus behind writing this paper. Whereas the seven other members of our team had extensiveexperience with pet therapy on various inpatient units at CAMH. Our varied experiences with pet therapy and the ways in which these experiences have influenced our thinking were the topic of many collaborative analytical conversations.

## Results

**Fig 1:**
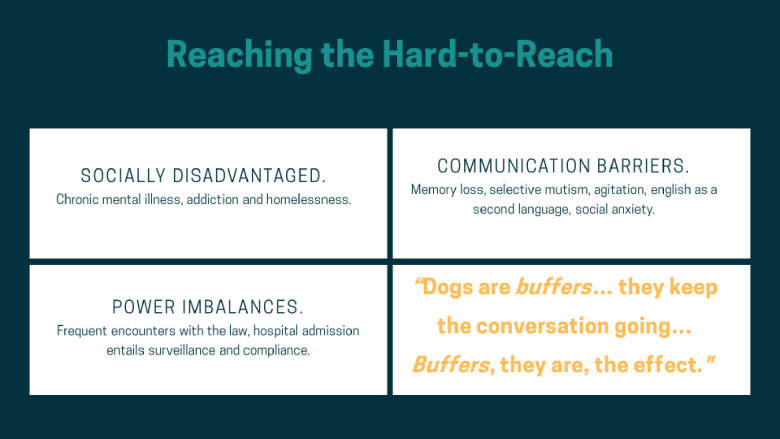
Reaching the hard-to-reach.

Rich and detailed qualitative data is only possible if participants are both *willing* and *able* to share their experiences. Our study demonstrated that the presence of a certified therapy dog increased many patients’ motivation to participate in our study, improved the ability of researchers to build rapport with participants and helped create a safe and open atmosphere that enabled patients to connect with each other and researchers. In short, therapy dogs bridge the gap between hard-to-reach populations and researchers, enabling research participants to share their lived experiences in a group setting (see Fig 1). We discuss each of these findings in more detail below.

### Motivation

Patients with depression, schizophrenia and dementia in particular often lack the **motivation** to complete everyday tasks. [24] In our study, the therapy dog helped counter-act the tendency of patients with mental illness to self-isolate. [25] For example, on the day of our FGD in the schizophrenia unit a patient that had expressed an interest in participating was having what was described by the RT to be a “a very bad day”. The researcher and the handler found him curled up in a ball in his room; he had not eaten or bathed all day. The dog jumped up on the bed and the patient reached out to pet and whisper something to him. A few minutes later he joined us for the FGD and later commented on how meaningful that brief one-on-one interaction with the dog was for him. In particular, patients enjoyed the opportunity to touch and be touched by another living being, something that was not always possible with fellow patients or staff. For example:

“They make you feel loved… When this dog licked my hand, it’s wow.” [Forensics]

Many participants also reported feeling more joyful and energized because of the dog’s presence. For example:

“I was feeling very down before he came, but now I’m: ‘George Michael, wake me up…’ Energized.” [GAU-B].

“I’m so excited. He [the dog] is the reason I didn’t stay in my room after group [therapy]. I was going to go to sleep. But I kept asking, where’s the dog? Where’s the dog?” [WIU].

“It [pet therapy] means I’m going to have a really good time.” [Acute Care -Schizophrenia].

RTs echoed these sentiments by noting that many asocial patients will leave their rooms to interact with a dog.

### Rapport

Building **rapport** is also critical to the success of qualitative research. Rapport is often established through the reciprocal exchange of neutral information about the weather, food or other shared experiences, or what we typically call “small talk”. [26] In the recruitment phase of this research, “small talk” with patients using the usual standbys, like “what brings you here?”, “how’s the food?” and “it’s so nice outside today, isn’t it?” would be inappropriate given that most in-patients have very limited access to the outdoors. The inability to create small talk in the typical manner can make it difficult to build rapport and create a common ground with research participants. However, talking about the dog was always appropriate. For example:

“It helps break the ice… With a dog around there is always something to talk about, right, and it’s not awkward. Maybe that’s what it is, it’s not awkward.” [WSFU].

“Since the dog died my wife and I have been hollering at each other, because [dogs], they’re *buffers*. They keep the conversation going…. *Buffers*, they are, the effect” [Emphasis ours, GAU-A].

We regularly engaged in simple conversations about the dog’s size, age, breed, training, behavior, and personality. It was through these routine exchanges of neutral information that we established a comfortable and open atmosphere that helped buffer the power gap between researchers and patients. For example:

“You just want to feel regular, because the pets do not make you feel as if you’re a patient. They treat you different… Like a human being.” [Forensics].

“So maybe it [dogs] helps people get along with other people better. It makes people better socializing with adults or other people their own age.” [WSFU].

The ability to build rapport with patients in a way that made them feel “like a human being” was critical to our success since it enabled us hold thoughtful discussions with a range of participants experiencing acute symptoms of mental illness.

### Connection

In this context, we were also working with vulnerable and socially disadvantaged populations who had many negative and sometimes violent experiences with the general public and/or encounters with the law. [27] For example:

“The thing that brightens up my day everyday if I’m out there panhandling and there are cops… but seeing someone walk by with a really cute dog and they let me pet it.. I like dogs more than people.” [GPU].

Many participants also described feeling isolated, dismissed and forgotten during most of their everyday social interactions. For example:

“He [the dog] listens. At least he listens. He’s not like most people, just ignore me.” [GAU-A].

Several participants also explained that they enjoyed pet therapy because they received so few visitors during their in-patient hospitalization. For example:

“I think it’s a way of *connecting* because people have their limits. You can’t walk up to somebody and comb their hair. So the animals provide an outlet.” [WSFU].

“If you don’t speak to anyone, you don’t socialize, you don’t go anywhere, by interacting with an animal you become more open to doing things with other people because you have the trust factor. *You feel more comfortable interacting with another person through pet therapy*. *It will help you open up*.” [Forensics].

Moreover, participants described their daily lives as inpatients as entailing a tremendous amount of compliance with hospital rules and regulations. These interpersonal dynamics can make it difficult for researchers to overcome the power imbalances that exist between researchers and participants. [28] In our FGD, the therapy dog clearly helped us **connect** to patients through the dog. Many participants described feeling more open and trusting with the therapy dog present. Petting and interacting with the dog also helped humanize these encounters and brought comfort to many patients in the midst of a mental health crisis.

More importantly, we found that the relaxed and open atmosphere created by the dog’s presence meant that participants more easily connected to their own experiences and memories. Without prompting, many participants told us detailed stories about their life histories. Many participants explicitly connected the dog’s presence to specific memories:

“I have always been around animals and worked on a farm, so it has been a good connection for me, memories… The memories it brings back,” [GPU].

“It reminds me of when I was on the outside in the neighborhood, even when I was a little guy… It reminds me of that and it’s a good feeling.” [Forensics].

Also, during the recruitment phase of this project we were chatting amiably for at least an hour with a group of participants during their regular pet therapy visit. One young man visibly enjoyed the dog and spent a great deal of time petting him. About thirty minutes into our visit he began to describe his entry into drug addiction. He started by telling us that he had to leave his dog behind when he went out West to find work. He described to us how lonely and boring this work camp had been. His counsellor told us afterwards that it was the first time she had ever heard him talk for any length of time. This effect was notable on every unit and was particularly useful for helping us prompt participants with memory problems (e.g. dementia) or help agitated participants focus on the topic at hand.

Participants routinely described the dog’s presence as a comfort that helped them navigate their social interactions with researchers and their fellow focus group participants more successfully. For example:

“The [dog] is opening everybody’s heart up.” [WIU].

“For me, I would say it’s comforting… because they don’t speak and I find I’m communicating with them on a different level. It’s different in a good way… They’re usually very friendly and you enjoy being with them, which is nice, and you feel a sense of connection.” [GPU].

“I think a lot of people are actively trying to deal with their personal boundaries, and it’s not that they’re too broad, sometimes people are too uptight. And I know I am one of those people.” [WSFU].

As experienced researchers, we can think of no other mechanism that so quickly and easily created an environment of trust and reciprocity.

## Discussion

**Fig 2:**
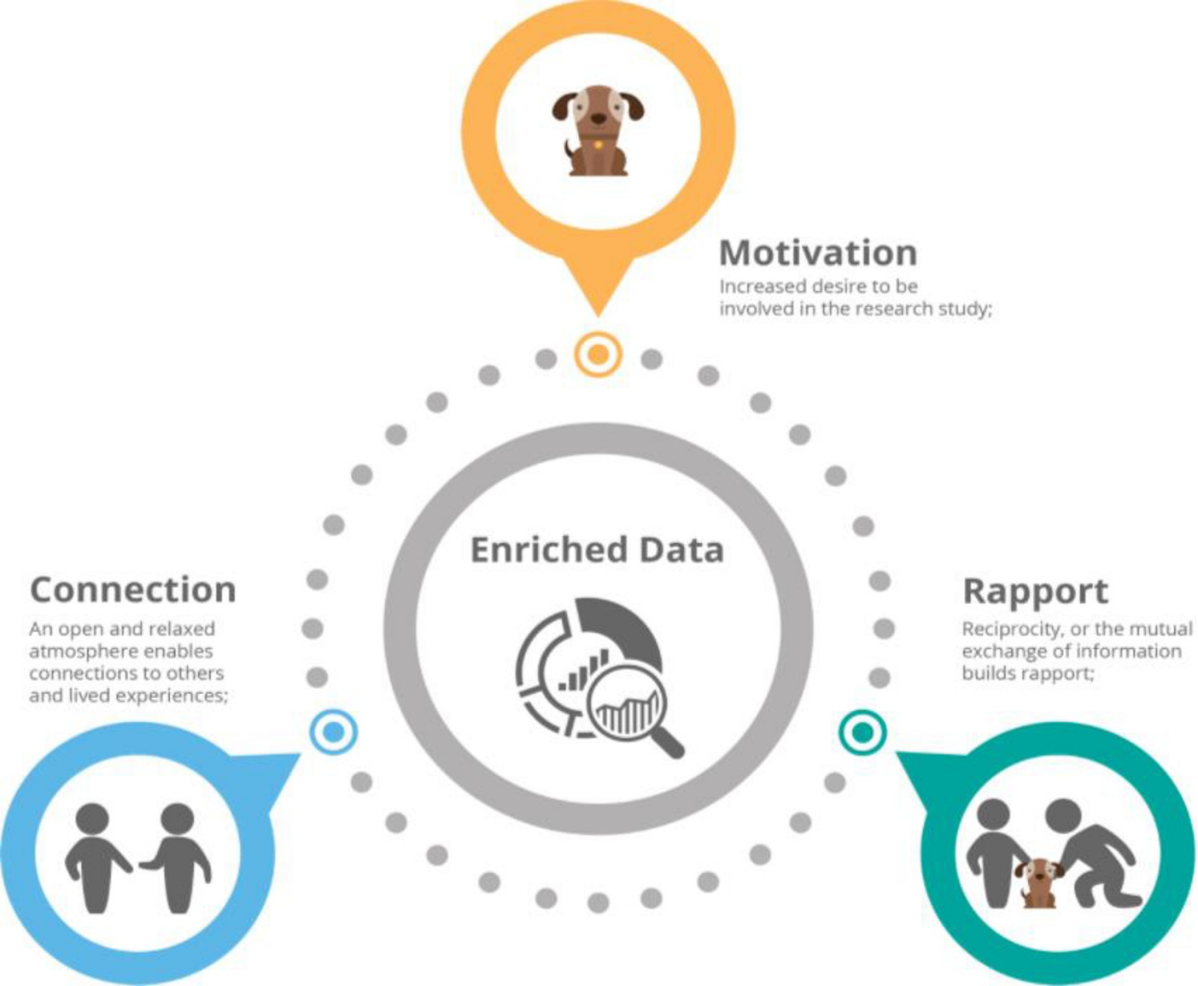
Increasing participation in research with certified therapy dogs.

Although our participants were hospitalized for acute symptoms of mental illness, nearly all were able to clearly articulate their likes and dislikes about pet therapy in a group setting. Our findings show that the presence of a therapy dog improved the depth and quality of the data we collected (see Fig 2). The dog served as a buffer by decreasing the power imbalance between the researchers and participants. In particular, therapy dogs add value in settings where it is difficult to build rapport or find common ground and where people may struggle to feel comfortable with individuals in authority. Similar to Kate Fox’s observations that “weather speak” is a form of code that enables people to overcome any natural reserve and talk to eachother; [29] “dog speak”, or conversation about a dog’s age, temperament and breed, helped us build rapport with our participants, which ultimately enriched our data. Significantly, the benefits of a certified therapy dog may also hold for individuals who do not enjoy dogs. For example, one of our participants’ did not like dogs but stated that she attended every pet therapy visit because they enjoyed the social interactions catalyzed by the dog’s presence. [30] In fact, during our FGD she kept herself behind a table so that the dog could not come up and interact with her, but told us she enjoyed the atmosphere generated by the dog’s presence.

Although there is a very large body of scholarship that explores the potential of various technological innovations to support patient engagement almost no attention has been paid to the role that animals, especially certified therapy dogs, might play at mediating social interactions during patient engaged research. An emerging body of research on interspecies interactions suggests that human and non-human actors mutually perceive and affect one another in particular material and social contexts. [27,31,32] For example, Solomon adapts Levinson’s (2006) metaphor of the “interaction engine” to argue that certified therapy dogs guide humans into rich social interactions by increasing their communication and affective ties with others (2008: 149). [33] McNicholas and Collis (2000) also illustrate how dogs enhance human well-being by strengthening social ties between people. [30] By paying attention to these dynamics our study highlights the benefit of using therapy dogs as a no-tech and low-cost alternative to buffer challenging social encounters and enhance the richness of qualitative data.

## Conclusion

Our qualitative study indicates that the presence of a therapy dog during our research activities increased the *motivation* to participate in our study, helped to build *rapport* with participants and created *connections* that enriched our data. These findings indicate that certified therapy dogs could be used effectively to engage participants in research about their treatment and care in a diverse range of medical settings. The ability of dogs to help our participants connect to their own memories and stay focused also suggests that therapy dogs might also be useful in clinical practice when taking medical histories or when discussing sensitive topics or traumatic events. [13] Further research on the impact of certified therapy dogs in clinical settings has not only the potential to humanize hospitalization of patients, but also to contribute to redressing some of the power imbalances that exist within research encounters.

## Supporting information

S1 Appendix Focus group discussion guide. (DOCX)
