# Supplementary material for: Increasing participation in research with therapy dogs: A qualitative study at a large urban mental health and addiction hospital
Source: PLoS One. 2020 Aug 27;15(8):e0238096. doi: 10.1371/journal.pone.0238096 (PMC7451510; doi:10.1371/journal.pone.0238096)
Supplement: S1 Appendix — (DOCX) [file pone.0238096.s001.docx]

**S1 Appendix A: Focus Group Discussion Guide**

1. Let’s start with a really broad question. What does pet therapy mean to you?
2. What interests you about pet therapy?
3. How do you feel when you feel when you hear that a dog is coming to visit?
4. How do you feel after the visit?
5. What do you think of the name “pet therapy”?
   - Probes: Is this is a good name for it? Why or why not? Is there a better name for it that you can think of?
6. What kinds of activities have you participated in during your pet therapy sessions? [Make a list on a flip chart].
7. What kinds of activities would you like to see more/less of?
8. Have you experienced any barriers to participating?
   - Probe for any possible negative experiences.
   - Examples could include: too many people, accessibility, (dog is on ground and hard to pet), distractions (e.g. tv on, noise, clashes with other planned activities, and so on)
9. How do you think pet therapy could be improved?
   - What kinds of challenges do you envision incorporating the suggested activity into these sessions? [can probe for multiple answers]
   - What do you think would be the benefits of this approach/new activity?
10. Is there any activity would you like to see included during pet therapy that we could try out now?
    - If they have suggested something to try, and we are able to do it, let’s do it at the end of the session;

**S2 Appendix: Participant Observation Guidelines**

Behaviors to observe during focus group discussions:

1. How do people talk to the dog? Do they use “Motherease” (e.g. talk to the dog like a small child)
2. Is the dog’s presence soothing or exciting? How does the dog’s mood change the atmosphere on the unit? How long do these mood changes last?
3. What kinds of social interactions does the animal facilitate?
   1. Does the dog facilitate interactions between patients? If so, what kinds of interactions?

| **Things to look for specifically** | **Questions to answer** |
| --- | --- |
| **Communication** |  |
| How do people talk to the dog? In what tone? (e.g. do they use “motherease” – a tone of voice used with children) |  |
| **Social Interactions** | What can we learn from popular discourse, institutional texts and everyday practices about:  The concept of fairness more broadly;  Competing or emerging definitions of fairness;  Fairness as it relates to health equity; |
| *Affect* |  |
| Verbal expressions |  |
| Physical expressions |  |
|  |  |
| **Atmosphere** | What can we learn about AI/ML as a *sociotechnical* system? |
| How is data collected and by whom? |  |
| How are workflows adapted and managed to accommodate data collection processes? |  |
|  |  |

| 1. **Patterns of decision-making** | How and when are datasets, declared accurate, unbiased and representative? And by whom? |
| --- | --- |
| *Gender, social networks and prestige hierarchies* |  |
| How and where do staff meet formally? Who is included/excluded from these gatherings? |  |
| How and where do staff meet informally? Who is included/excluded from these gatherings? |  |
| To what extent do social networks, gender and prestige hierarchies shape decision-making processes? |  |
|  |  |
| How, when and where to data curators, clinicians and patients intersect? |  |
| **Cultural ideals [social constructions of ]and fairness** | What can we learn from popular discourse, institutional texts and everyday practices about:  The concept of fairness more broadly;  Competing or emerging definitions of fairness;  Fairness as it relates to health equity; |
| *Fairness* |  |
| What is the public discourse around fairness? Who uses the term and in what context? |  |
| What are people’s attitudes towards the social determinants of health, equity, and social justice? |  |
| How are health priorities determined? |  |
| **Data Collection Processes** | What can we learn about AI/ML as a *sociotechnical* system? |
| How is data collected and by whom? |  |
| How are workflows adapted and managed to accommodate data collection processes? |  |
|  |  |

- 1. Dog as communication conduit
